# Proteome analysis of human Wharton's jelly cells during *in vitro *expansion

**DOI:** 10.1186/1477-5956-8-18

**Published:** 2010-03-26

**Authors:** Stefania Angelucci, Marco Marchisio, Fabrizio Di Giuseppe, Laura Pierdomenico, Marilisa Sulpizio, Enrica Eleuterio, Paola Lanuti, Giuseppe Sabatino, Sebastiano Miscia, Carmine Di Ilio

**Affiliations:** 1 Department of Biomedical Science, G d'Annunzio University, Chieti-Pescara, Italy; 2 Center of Excellence on Ageing, G d'Annunzio University Foundation, Chieti, Italy; 3 Department of Biomorphology, G d'Annunzio University, Chieti-Pescara, Italy; 4 Neonatology Unit, Department of Ageing Sciences, "G d'Annunzio" University, Chieti-Pescara, Italy; 5 Stem TeCh Group, Chieti, Italy

## Abstract

**Background:**

The human umbilical cord contains mucoid connective tissue and fibroblast-like cells. These cells named Wharton's jelly cells, (WJCs) display properties similar to mesenchymal stem cells therefore representing a rich source of primitive cells to be potentially used in regenerative medicine.

**Results:**

To better understand their self-renewal and potential *in vitro *expansion capacity, a reference 2D map was constructed as a proteomic data set. 158 unique proteins were identified. More than 30% of these proteins belong to cytoskeleton compartment. We also found that several proteins including Shootin1, Adenylate kinase 5 isoenzyme and Plasminogen activator-inhibitor 2 are no longer expressed after the 2^nd ^passage of *in vitro *replication. This indicates that the proliferative potency of these cells is reduced after the initial stage of *in vitro *growing. At the end of cellular culturing, new synthesized proteins, including, ERO1-like protein alpha, Aspartyl-tRNA synthetase and Prolyl-4-hydroxylase were identified. It is suggested that these new synthesized proteins are involved in the impairment of cellular surviving during replication and differentiation time.

**Conclusions:**

Our work represents an essential step towards gaining knowledge of the molecular properties of WJCs so as to better understand their possible use in the field of cell therapy and regenerative medicine.

## Background

Since the first identification of non-hematopoietic stem cells in the bone marrow as colony-forming unit-fibroblasts (CFU-Fs) and the detailed characterization and description of the tri-lineage potential of the mesenchymal stem cells (MSCs), our knowledge of the molecular properties of these cells has made great progress [1,2]. MSCs have a great appeal for tissue engineering and therapeutic applications because of their high *in vitro *expansion potential, self renewal capacity and multi-potentiality [3-5]. However, considering the invasive procedure related to their availability, there is an increasing interest in investigating the presence of MSCs in adult and fetal sources and especially their presence in fetal membranes such as umbilical cord matrix [6-9].

**Figure 1:**
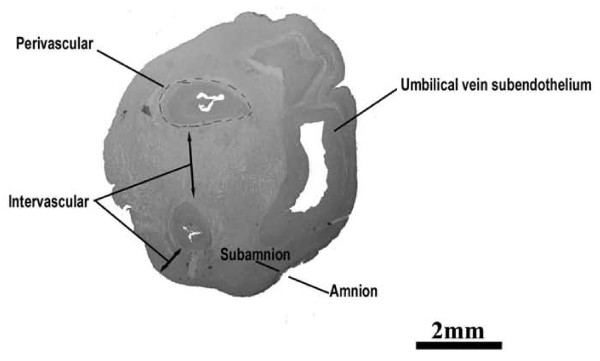
**Umbilical Cord Compartment**. Digital photo of human umbilical cord tissue. Traverse section through an umbilical cord after birth. Scale bar = 2 mm. Wharton's Jelly is the connective tissue included between the subamnion and the perivascular regions.

In the umbilical cord two arteries and one vein, surrounded by mucoid connective tissue, called Wharton's jelly are present (figure 1). It contains fibroblast-like cells (WJCs), which show properties similar to the MSCs and may represent a rich source of primitive cells [10,11]. Like bone marrow stromal cells, WJCs are plastic adherent, positively stained for markers of the mesenchymal cells and negatively stained for markers of the hematopoietic lineage [10-14].

WJCs can be expanded longer than MSCs before its multeplicity is compromised [11]. For this reason they could be induced to form different cellular lineages i.e. adipose tissue, bone, cartilage, skeletal muscle cells, cardiomyocyte-like cells and neural cells enabling them to be used in biomedical engineering applications [15-17].

The aim of the present work was to build a 2D reference map as a proteomic dataset, useful to define the molecular characteristics of WJCs and also to discover a number of candidate biomarkers specifically expressed in these extra-embryonic stem cells. In addition we also studied the molecular change that occurs during the WJCs *in vitro *growth in order to find proteins possibly related to their long expansion ability and fast growth *in vitro*.

## Methods

### Isolation and cell culture

For cell culture institutional review board approval was obtained for all procedures. With the consent of the parents, fresh human umbilical cords were obtained from full-term births, aseptically stored in sterile saline and processed within 6 hours from partum to obtain the umbilical cord matrix mesenchymal stem cells. After removal of blood vessels, the abundant extracellular matrix of Wharton's jelly was scraped off with a scalpel, finely cut and centrifuged at 250 × g for 5 minutes at room temperature and the pellet was washed with serum-free Dulbecco's modified Eagle's medium (DMEM). Next, the cells were treated with collagenase (2 mg/ml) (Sigma) for 16 hours at 37°C, washed in PBS(1X), and treated with 2.5% trypsin for 30 minutes at 37°C under agitation (Wang, Sarugaser). Finally, the cells were washed in PBS(1X) and seeded in complete growth medium (HMSCGM) with growth supplements, all from Cambrex Bio Science (now Lonza, Walkersville, Inc., Walkersville, MD), in 5% CO_2 _in a 37°C incubator (ref Sarugaser, Weiss, Mitchell). The cells were cultured with the medium exchange every 3-4 days until reaching the confluence. The adherents cells were detached with 0,05% trypsin-EDTA, counted with Trypan Blue exclusion, and re-seeded at 3000 cells/cm^2 ^to reach the 90% of confluence after 3-4 population doublings.

### Immunophenotype

The WJCs from Wharton's jelly after the harvesting at the different experimental times (2^nd^, 4^th^, 8^th ^and 12^th ^culture passages) were immediately treated with 0.05% trypsin-EDTA and incubated with 1 μg/10^6 ^cells fluorescein isotiocynate (FITC)-conjugated or phycoerythryne (PE)-conjugated antibodies for 40 minutes at 4°C in the dark. The anti-CD73, anti-CD13, anti-CD90, anti-CD117, anti-CD14, anti-CD34, anti-CD105 and anti-CD45 (Becton Dickinson, San Jose, CA, USA), anti-CD29, anti-CD44 and anti-CD166 (Ancell, Bayport, MN, USA) antibodies were used. After washing, cells were analyzed on a flow cytometer (FACSCalibur, Becton Dickinson, San Jose, CA, USA) collecting 10,000 events and the data analyzed by Cell Quest Software (Becton Dickinson, San Jose, CA, USA).

### Cell cycle

5 × 10^5 ^cells/sample for each passage were fixed by adding 500 μl of 70% cold ethanol and then stored at 4°C. After 24 h cells were washed and stained by adding 500 μl of a solution containing 50 μg/ml propidium iodide (PI) and 200 μg/ml RNase. Cells were incubated for 3 h at 4°C in the dark and acquired on a FACSCalibur flow cytometer using the CellQuest™ software (both from Becton Dickinson, USA). Debris was excluded from the analysis by gating a forward scatter versus side scatter plot. Cell aggregates were excluded by gating FL2 area versus FL2 width. The low flow rate mode (about 400-500 events/second) was used to record 10,000 non-debris events for each sample. PI fluorescence data were collected using linear amplification. DNA content was assessed, placing the G1 peak around the channel 400. Finally, data were analysed using ModFit LT™ software (Verity Software House, Toshan, ME, USA).

### Telomere length assay

DNA extraction was performed using Wizard Genomic DNA Purification Kit (Promega) following the manufacturer's instructions. The length of telomere regions of genomic DNA was assessed on DNA from cells at different passages using the Telo TAGGG kit (Roche) according to the manufacturer's instructions. Appropriate controls, DNA extracted from cells with long or short telomere regions, were also provided with the kit.

### Determination of cell senescence

The amount of senescent cells was evaluated at different conditions by the use of the Senescence β-Galactosidase Staining Kit (Abcam, Cambridge, UK) in accordance to the manufacturer's instructions. Thus, the cells at different experimental conditions (2^nd^, 4^th^, 8^th ^and 12^th ^passage) were plated a density of 10,000 cells/cm^2 ^for 24 h before senescence-associated β-galactosidase staining. After completion of the staining procedures, 5 representative images were taken from diverse areas of each cell culture, using phase-contrast microscopy to asses the number of positive cells.

### Adipogenic differentiation

To induce adipocyte differentiation, 20 × 10^3 ^cells/cm^2 ^was cultured in DMEM high glucose (HG) (Sigma) supplemented with 10% FBS, 0.5 mM isobutyl-methylxantine (Sigma), 200 μM indomethacin (Sigma), 1 μM dexamethasone (Sigma) and 10 μg/ml insulin (Sigma). The cells were cultured, replacing the medium every 2-3 days. After 2-3 weeks of culture, the cells contained neutral lipids in fat vacuoles; they were fixed in 10% formalin and stained with fresh oil red-O solution (Sigma).

### Osteogenic Differentiation

To induce osteogenic differentiation, 3 × 10^3 ^cells/cm^2 ^was cultured in MEM (Sigma) supplemented with 10% FBS, 10 mM β-glycerophosphate (Sigma), 0.2 mM ascorbic acid (Sigma), and 10 nM dexamethasone (Sigma), and cultured for 3-4 weeks, replacing the medium every 2-3 days. To demonstrate osteogenic differentiation, the cultures were fixed and subjected to alkaline phosphatase.

### Chondrogenic Differentiation

To induce chondrogenic differentiation, aliquots of 2.5 × 10^5 ^cells were pelleted in polypropylene conical tubes in 0.5 ml of DMEM HG containing 6.25 μg/ml insulin (Sigma), 6.25 g/ml transferrin (Sigma), 6.25 g/ml selenous acid (Sigma), 5.33 μg/ml linolenic acid (Sigma), 1.25 mg/ml BSA (Sigma), 0.35 mM proline (Sigma), 1 mM sodium pyruvate (Sigma), 100 nM dexamethasone (Sigma), 0.1 mM L-ascorbic acid-2-phosphate (Sigma), supplemented with 10 ng/ml transforming growth factor-beta 3 (TGF-β3) (R&D Systems, Minneapolis, MN). This medium was replaced every 3-4 days for 3-4 weeks. Pellets were formalin-fixed, embedded in paraffin, morphologically examined after toluidine blue staining.

### Proteome characterization

To generate 2D map of WJCs and analyze their protein changes during culture condition we chose four passages (2^nd^, 4^th^, 8^th ^and 12^th^) *in vitro *expansion that were processed for protein extraction. For all culture passages we considered three independent donors and we ran 3 gels for each biological replicate. 16 × 10^6 ^stem cells of each population were treated with lysis buffer (40 mM Tris pH 7.4, 8 M urea, 4% CHAPS) supplemented with protease inhibitor mixture and 2 mM TBP as reducing agent. Protein concentration was determined by using the Bradford assay with bovine serum albumin (BSA) as standard [18].

The lysated samples were loaded onto commercial 4-7 IPG strip and the second dimension was performed on a 9-16% acrylamide gel. Analytical gels were stained with ammoniacal silver nitrate, while gels used for MALDI-TOF MS protein identification were silver-stained without glutaraldehyde, according to the mass compatible method described by Shina et al. [19,20]. After staining, gels were scanned with a MagicScan scanner (GE Healthcare, formerly Amersham Biotech, Uppsala, Sweden) in transparency mode at 800 dpi and the images were stored as TIFF images. Once digitized, the gel images were analyzed with Image Master 2D platinum software, 6.0 versions (GE Healthcare, formerly Amersham Biotech, Uppsala, Sweden). A positional gel calibration was carried out by using a 2D calibration method included in the analysis package that calculates the position of protein spots in terms of their isoelectric point (pI) and Molecular Weight (MW) values. Several spots, Thioredoxin (pI 5.13-47.09 kDa), Endoplasmin (pI 4.60-90.13 Da), spectrin alpha chain (pI 5.6-118.17 kDa), 75 kDa glucose regulated protein (pI 6.2-74.02 kDa), Glycerol-3-phosphate dehydrogenase (pI6.1 -38.65 kDa), distributed throughout the reference gel and identified by mass spectrometry analysis were chosen as marker proteins. Then a protein list including these marker proteins was created and pI and MW were calibrated in all maps simultaneously by interpolating between the known pI and MW values of the markers. In order to construct a synthetic gel from all conditions included in the present study three different gel runs for each sample were performed and then subjected to image analysis with Image Master 2D evolution software (Ge Healthcare, formerly Amersham Biotech, Uppsala, Sweden). A spot was considered only if it was detected in three out of all experimental replicates for each sample and defined "common spot" (i.e. always present and in the same position on all gels). The quantity of each spot was normalized with respect to the total spot volume detected in the gel. Relative spot volumes were determined by modelling the optical density in individual spot segments using a two-dimensional Gaussian analysis.

Following gel analysis, protein spots on preparative WJCs 2-DE gels were excised and analyzed by the peptide mass finger printing (PMF) approach on a MALDI-TOF MS. Protein spots, once excised from the gel, were washed with 100% ethanol and 100 mM ammonium bicarbonate (NH4HCO3). The pieces of gel corresponding to a single protein were incubated for 60 min at 56°C in a volume of 50 mM NH4HCO3 supplemented with 10 mM DTT and then for 30 min in the dark in 55 mM iodoacetamide in 100 mM NH4HCO3 at room temperature. Finally, the gel was reswollen in 50 mM NH4HCO3 containing trypsin and incubated at 37°C overnight [21]. Peptide extract was applied to a C18ZipTip (Millipore, Bedford, MA, USA), rinsed with a 0.1% TFA and eluted directly on the MALDI target with 0.5 μl of a saturated α-cyano-4-hydroxycinnamic acid (1:1 = ACN: 0.1% TFA) solution.

For all MS analysis, PMF was obtained using REFLEX-IV MALDI-TOF (Bruker Daltonics, Germany) in the reflectron operation mode at a potential of 20 kV and a delayed extraction of 400 ns. The instrument was calibrated with external standards such as bradykinin (fragment 1-7) 757,39 m/z, angiotensin II 1046,54 m/z, ACTH (fragment 18-39) 2465,19 m/z, Glu Fibrinopeptide B 1571,57 m/z, and renin substrate tetradecapeptide porcine 1760,02 m/z. Each spectrum was produced by accumulating data from 100 laser shots over the m/z range of 700-3000 Da. All mass spectra were calibrated internally by trypsin (Promega Bioscience, CA) autolysis products at 842, 50 Da, 1.046,56 Da, 2.212,11 Da and 2.284,19 Da. Trypsin and keratin contamination peaks were excluded from the peak list used in the database searching.

The results from the PMF were employed to search the human NCBIn protein database by Mascot search engine, which compare the experimentally determined tryptic peptide masses with theoretical peptide masses calculated for protein from these databases. Search parameters are as follows: type of search, peptide mass fingerprint; enzyme, trypsin; fixed modification, carbamidomethylation (Cys); variable modifications, oxidation (Met); mass values, monoisotopic; peptide charge state, 1+; maximum missed cleavages, 1; and peptide mass tolerance, 100 ppm.

Confirmation of the proteins expressed by WJCs at the 2^nd ^and 12^th ^culture passages was made by Western blot analysis in the 1D and 2D mini system. WJCs samples at 2^nd ^and 12^th ^passages were extracted and centrifuged. An equal protein amount of 10 μg for 1D from each total cellular lysate was loaded into 9-16% SDS-PAGE and was electrophoretically transfererred into nitrocellulose membranes at 100 V overnight at 4°C. For 2D Western blot 20 μg of WJCs cells at 2^nd ^and 12^th ^passages were separated on 7 cm pH 4-7 IPG strip (GE Healthcare, formerly Amersham Biotech, Uppsala Sweden). IEF was performed by a 12 h in gel-rehydratation mode at 50V, followed by focusing at 5000 V for a total of 20 kVh. The strips were incubated in equilibration buffer (8 M urea, 2% SDS, 30%(v/v) glycerol, 50 mM Tris-HCl, pH8.8) reduced with 1% (w/v) DTT for 15 min and alkylated with 2.5% iodacetamide for 15 min in dark. The 2D running was made into 9-16% SDS-PAGE and the electrophoresed proteins were transferred to nitrocellulose at 200 V overnight at 4°C. Primary antibodies sources and dilutions were: rabbit polyclonal antibodies against ADK5 1:100 (Abcam, Cambridge Science Park, UK), PAI2 1:1000 (Abcam, Cambridge Science Park, UK), SHOT1 1:500 (Cell Signalling Technology inc., Denver, MA), ERO1A 1:1000 (Abcam, Cambridge Science Park, UK), P4HA1 1:1000 (Abcam, Cambridge Science Park, UK), P4HA2 1:1000 (Abcam, Cambridge Science Park, UK), VIME 1:1000 (Abcam, Cambridge Science Park, UK), chicken polyclonal antibody against EIF3I 1:2000 (Abcam, Cambridge Science Park, UK), and mouse monoclonal antibody against ACTB 1:1000 (Santa Cruz Biotechnology, Santa Cruz, CA). Secondary antibodies diluted 1:6000 were goat polyclonal to rabbit IgG (for ADK5, PAI2, SHOT1, ERO1A, P4HA1, P4HA2 and VIME) rabbit polyclonal to chicken IgY (for EIF3I) and goat polyclonal to mouse IgG (for ACTB), all conjugated with horseradish peroxidase (Abcam, Cambridge Science Park, UK). Signal detection was performed with an ECL plus kit (GE Healthcare formerly Amersham Biotech, Uppsala, Sweden) and visualized by autography on Biomax light film (Sigma Chemical, St. Louis, MO, USA). All blot experiments were performed at least three times.

## Result and Discussion

### Cellular isolation, culturing and characterization

**Figure 2:**
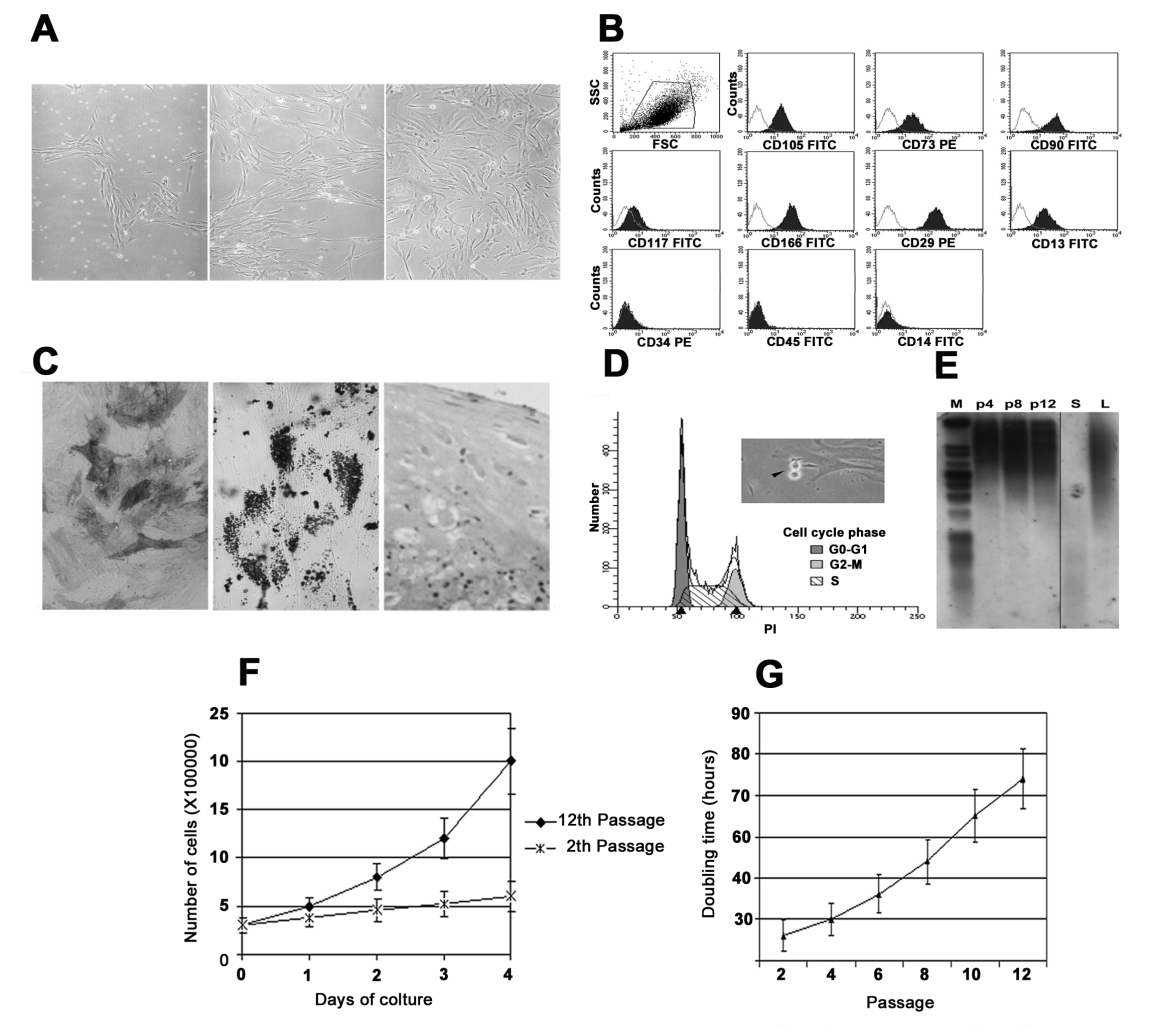
**WJCs characterization**. **A. *Light microscopic micrographs of WJCs in monolayer culture***. In monolayer culture, the cells assumed a polymorphic, fibrobroblast-like morphology, which was maintained throughout the passaging processes. 10 days (left panel), 15 days of culture (central panel) and cells at passage 1, prior to reach confluence (right panel). Original magnification ×100. **B. *Analyses of surface antigen marker by flow cytometry***. Cell suspensions were stained with specific mouse anti-human monoclonal antibodies (Mabs) as indicated in filled histograms. The empty histogram is the respective IgG isotype control. **C. Ability to differentiate into several lineages**. Osteogenic differentiation (left panel) was indicated by the increase in alkaline phosphatase (magnification 200×). Adipogenic differentiation (central panel) is visually marked by accumulation of neutral lipid vacuoles in cultures (red oil staining) (magnification 200×). Chondrogenic differentiation (right panel) is visually marked by toluidine blue staining (magnification 100×). **D. *Flow cytometry of cells showing DNA stained with propidium iodide***. G1 and G2/M indicate 2n and 4n cellular DNA content, respectively. S indicates cells undergoing DNA synthesis, intermediate in DNA content between 2n and 4n. In the phase contrast image, arrow indicates one mitotic event. **E. *Telomere length assay on WJCs at different culture passages***. The maintenance of long telomeres is a key feature of stem cells, ensuring the capability to undergo several cell cycles of replication. Molecular weight marker (M), control DNA with short telomeres (S) and control DNA with long telomeres (L). p4, p8 and p12 DNA extracts from WJCs at different 4^th ^8^th ^and 12^th ^passages showing long telomeric ends. **F, G. *Growth Features of WJCs during expansion period***. Proliferation (F) and doubling time (G) at different passages of WJCs in vitro culture calculated by Trypan blue exclusion test.

As can be seen in figure 2A, the cells isolated from the human Wharton's jelly and used in the present work, display a consistent spindle-shaped elongated morphology similar to fibroblastoid cells. This feature appears to be maintained up to the end of culturing. A representative immunophenotype of the cells used in our experiment is reported in figure 2B. A positive response for CD105 (endoglin), CD73, CD90, CD29, CD166, CD13, CD44 (hyaluronan receptor) markers and a negative reactivity for CD34, CD45 and CD14 hematopoietic markers were detected at each time of culture passages. This high homogeneous expression of the above mentioned markers suggests that the samples used in the present study were not significantly contaminated by no-stem cell population (figure 2B). Figure 2C illustrates the ability of WJCs to differentiate in osteogenic, adipogenic and chondrogenic lineages further evidencing their pluripotency.

As reported in figure 2D cells, after expansion, showed a homogeneous diploid content in the G1 cell-cycle phase. Furthermore, in figure 2D it can be also seen that the G1 and the G2 cell-cycle checkpoints appeared intact. This finding is consistent with cells actively cycling. The long telomeric end of DNA extracted from cells at all passages examined also confirms that these cells preserve their capability to undergo a high number of cellular divisions up to the 12^th ^passage (figure 2E). Accordingly, a low frequency of β-galactosidase positive staining cells was found in all different passages (2^nd^, 4^th^, 8^th ^and 12^th^) (Additional file 1). Overall, the data reported in figure 2 strongly indicate that the cells derived from Warthon's jelly belong to the mesenchimal stem-cell population.

In order to characterize the proteome profile of WJCs, the cells prepared at the 2^nd^, 4^th^, 8^th ^and 12^th ^culture passages were analyzed. Longer term *in vitro *culture passage led to an impairment of the cell expansion ability as accounted by the cell number count (figure 2F) and by the analysis of the doubling time (figure 2G). For these reasons they were not included in the present investigation.

### 2DE analysis and MS protein identification

**Figure 3:**
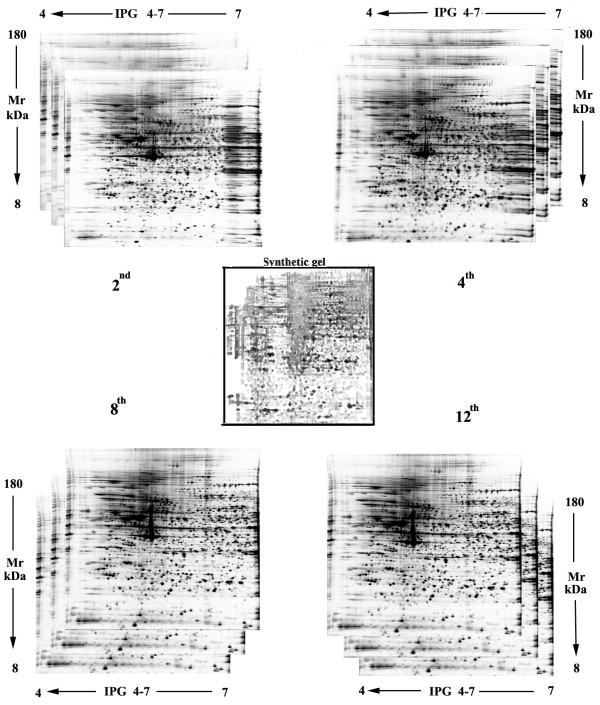
**WJCs 2D map**. Silver-stained 2D gels (pH4-7, 9-16% acrylamide, 200 μg protein loading) from the 2^nd^, 4^th^, 8^th ^and 12^th ^*in vitro *expansion passages. The synthetic gel is the result of the comparative analysis between the master gels obtained from 3 gels for each culture passage.

The master gels of the 2^nd^, 4^th^, 8^th ^and 12^th ^passage constructed for each step are reported in figure 3, by using cells prepared from three different human donors. On average 2450 ± 76, 2330 ± 91, 2200 ± 103 and 1750 ± 74 spots were detected from the 2^nd ^to the 12^th ^*in vitro *expansion passages respectively (Additional file 2).

In order to evaluate the change of protein pattern expression occurring during in *vitro *cell expansion, the master gels, resulting from each culture passages, were compared with the synthetic gel constructed by using the gels resolved from all culture passages (figure 3).

**Figure 4:**
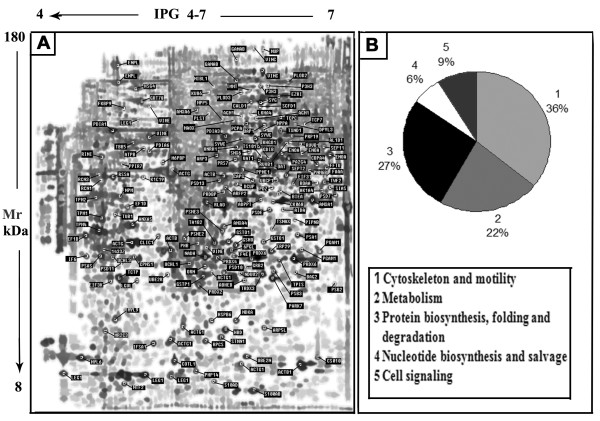
**WJCs shyntetic gel (A) and the functional grouping of identified proteins (B)**. In the Figure 4B values represent the percent distribution of proteins classified by biological process of gene ontology terms into functional categories.

Image analysis of the synthetic gel (figure 4A) revealed the presence of 2150 spots. Since these spots are common to all passages they can therefore be considered housekeeping proteins. 251 out of 2150 spots were picked for mass spectrometry analysis. 183 spots, representing 158 unique proteins with a molecular mass ranging from 12 kDa to 180 kDa and with an isoelectric point in the 4-7 pH range were identified. The list of assigned proteins are indicated in the 2D map (figure 4A) and reported in Additional file 3. The identified proteins could be included into at least five functional categories (figure 4B), i.e. cytoskeleton and motility, metabolism, protein biosynthesis folding and degradation, nucleotide biosynthesis and cell signaling. Overall 60% of total proteome have been identified as proteins belonging to the cytoskeleton compartment and involved in protein biosynthesis, folding and degradation. The great number of proteins of these two groups may probably give to WJCs the proliferative capacity for the quickly change of their phenotype in response to adequate external stimuli.

The large number of proteins associated with the energetic metabolism may confer to WJCs the necessary power for their intense proliferation. Antioxidant proteins such as thioredoxin, peroredoxin and gluthatione transferases, are also present in these cells. They may play a key role in preserving WJCs from oxidative injury during *in vitro *expansion.

Proteins involved in nucleotide biosynthesis and signal transduction seem to be poorly represented in WJCs. It has to be noted that myocyte's characterizing proteins like caldesmon, α-actinin, tropomyosin, and vinculin are expressed up to the last culture passages [22]. Furthermore cardiomyogenic and hepatic putative markers such as tropomyosin and pyruvate kinase were also detected in these cells [23-26].

**Figure 5:**
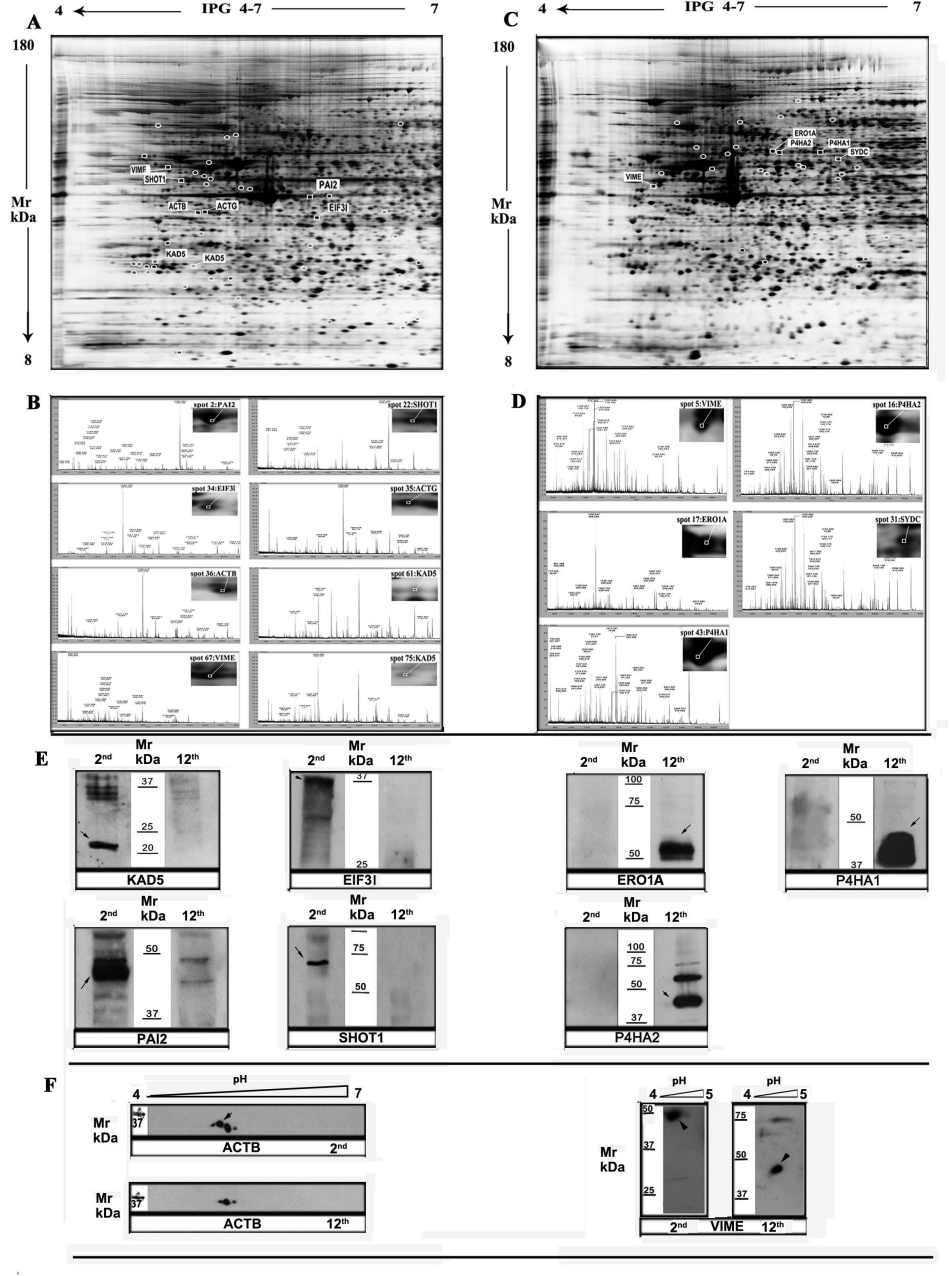
**Panel A and B indicate proteins no longer expressed after the 2^nd ^passage**. Protein spots detected by image analysis (**A**) are indicated with circles, whereas the proteins assigned by PMF analysis are annotated with the abbreviation name; the magnified views and peptide mass spectrum of spots 2, 22, 34, 35, 36, 61, 67 and 75 are indicated (**B**). **Panel C and D indicate proteins spots expressed at the 12^th ^passage.** All detected spots are indicated with circles, whereas the identified protein spots are designed with the abbreviation name (**C**); the magnified views and peptide mass spectrum of spots 5, 16, 17,31 and 43 are indicated (**D**)**. Panel E and F indicate immunoblot analysis of the WJCs protein only expressed at the 2^nd ^and at the 12^th ^passages**. 1-D gels probed with antibodies raised against KAD5, EIF31, PAI2, SHOT1, ERO1, PHA1 AND PHA2 (**E**). 2D blot analysis raised against ACTB and VIME at the 2^nd ^and 12^th ^passages (**F**).

The presence of vimentin (VIME), which is the most ubiquituos intermediate filament protein and the first protein to be expressed during cell differentiation is common to other MSCs such as bone marrow and cord blood derived non-hematopoietic (mesenchymal, stromal) progenitor cells, fibroblasts and endothelial cells [27,28]. VIME is one of the most prominent phosphoprotein present in MSCs and its phosphorylation is significantly enhanced during cell division [29,30]. Six different isoforms of phosphorilated VIME were identified in WJCs. However, one of them i.e. Vimentin 4.9/47.4 (table 1; figure 5F) was no longer expressed after the 2^nd ^passage, whereas another one i.e. Vimentin 5.1/45.7 (table 2; figure 5F) appeared only at the end of the last culture passage.

**Table 1:** Proteins no longer expressed after the 2^nd ^culture passage.

Spot #	Abbr. Name	AC^a^	Protein Name	Score^b^	SC^c ^%	pI	Mr(Da) Theo.	pI	Mr(Da) Exp.
2	PAI2	P05120	Plasminogen activator inhibitor 2	123	46	5,46	46596	5,44	30700
22	SHOT1	A0MZ66	Shootin 1	56	39	5,27	72109	4,80	50629
34	EIF3I	Q13347	Eukaryotic translation initiation factor 3 subunit I	214	61	5,38	36502	5,21	34955
35	ACTG	P63261	Gamma Actin	68	32	5,31	41792	4,98	32910
36	ACTB	P60709	Beta Actin	96	58	5,29	41736	5,04	33210
61	KAD5	Q9Y6K8	Adenylate kinase isoenzyme 5	81	37	5,23	22420	4,80	28831
67	VIME	P08670	Vimentin	100	35	5,06	53651	4,95	47492
75	KAD5	Q9Y6K8	Adenylate kinase isoenzyme 5	79	54	5,23	22420	4,70	27829

A) Entry code number from SWISS PROT database.

B) Score is -10*Log(P), where P is the probability that the observed match is a random event, it is based on Swiss-Prot database using the MASCOT searching program as MALDI-TOF data.

C)Sequence coverage means the ratio of portion sequence covered by matched peptide to the full length of the protein sequence.

**Table 2:** New proteins expressed after the 12^th ^culture passage.

Spot #	Abbr. Name	AC^a^	Protein Name	Score^b^	SC^c ^%	pI	Mr(Da) Theo.	pI	Mr(Da) Exp.
5	VIME	P08670	Vimentin	124	58	5,06	53651	5,09	45706
16	P4HA2	O15460	Prolyl 4-hydroxylase subunit alpha-2	64	53	5,50	61623	5,50	61292
17	ERO1A	Q96HE7	Oxidoreductin-1-Lalpha	98	71	5,43	55213	5,65	58342
31	SYDC	P14868	Aspartyl-tRNA synthetase, cytoplasmic	89	67	6,10	57700	6,09	57478
43	P4HA1	P13674	Prolyl 4-hydroxylase subunit alpha-1	101	45	5,70	61157	5,12	62401

A) Entry code number from SWISS PROT database.

B) Score is -10*Log(P), where P is the probability that the observed match is a random event, it is based on Swiss-Prot database using the MASCOT searching program as MALDI-TOF data.

C) Sequence coverage means the ratio of portion sequence covered by matched peptide to the full length of the protein sequence.

It is interesting to note that proteins reported to be markers of MSCs such as Annexin 1, Annexin 2, or heat shock protein 27 b (HSP27 b) and proteins considered markers for hESCs such as elongation factor Tu (TUFM), isocitrate dehydrogenase (IDH1), peroxiredoxins 1, 2, and 6 (PRDX1, PRDX2, PRDX6) are simultaneously expressed in WJCs [31]. Furthermore, WJCs contain nuclesphosmin, a nucleolus protein, which is highly expressed in proliferating cell as well as in undifferentiated hESCs [32-35]. Two signal transduction proteins, i.e. 14-3-3 protein zeta/delta and Twinfillin-like protein 2, not yet found in any of the stem cell lines so far investigated, are present in WJCs. The possibility that they may be WJCs specific proteins needs to be further investigated. The gel matching between the master gels and the synthetic gel has allowed to detect 47 proteins that were no longer expressed after the initial passages (figure 5A).

8 out of 47 were assigned by PMF (figure 5B) and six of them were validated by Western blotting experiments (table 1 and figure 5E, F). These proteins are involved in important cellular physiological functions including protein biosynthesis, cellular division, internal cellular motility and neuronal differentiation.

Shootin 1 (SHOT1) and two Adenylate Kinase 5 isoenzymes (KAD5) have been identified as brain specific proteins that may play a key role in neurogenesis [36-38]. The eukaryotic translation initiation factor 3 subunit 1 (EIF3I) has an essential role in the rate-limiting initiation phase of translation and is required for several steps in the initiation of protein synthesis [39]. Although the physiological role of Plasminogen activator inhibitor 2 (PAI2) has not yet been well defined, several intracellular functions, including capacity to alter genes expression, ability to influence the cellular proliferation and differentiation or inhibition of apoptosis, have been attributed to this protein [40,41].

The inability of WJCs to express, after the initial stage of replication, specific proteins involved in neuronal differentiation, may indicate that WJCs are progressively reducing their differentiation capacity during *in vitro *expansion.

Gel matching analysis has also allowed to identify 27 new proteins that appear only at the end of culturing (figure 5C).

5 out of 27 (table 2) were assigned by PMF (figure 5D) including two isoforms of Prolyl-4-hydroxylase i.e. the subunit alpha-1(4PHA1) and the subunit alpha-2 (4PHA2). Western blotting analysis was carried out on them and on ERO1-like protein alpha; the data obtained are shown in figure 5F.

4PHAs promote the post-translational formation of 4-hydroxyproline in hypoxia-inducible factor (HIF) alpha proteins which physiologically regulates a broad range of relevant cellular functions including apoptosis, cellular survival and energy metabolism [42]. 4PHAs are considered cellular oxygen sensors able to modulate the cellular response towards hypoxia [43-45].

In addition, these enzymes are involved in the intracellular collagen maturation by catalyzing the hydroxylation of proline residues of the procollagen. Their presence may probably be a consequence of the impairment of the cellular expansion ability that occurs to WJCs at the end of cellular *in vitro *expansion (figure 2F, G).

Accordingly, Miyaishi et al. have found that human fibroblasts at low population doubling rate increase the level of 4PHA1 and 4PHA2 expression [46].

At the end of culturing we have also found ERO1-like protein alpha (ERO1A). This enzyme is responsible for ROS cellular production during oxidative stress [47-49]. It is well known that ROS are considered the major contributors to cellular aging [50]. This protein, induced by hypoxia and regulated via HIF-pathway may play a key role in the maintenance of cellular redox homeostasis.

An interesting protein we have found to be expressed only at the end of culture expansion is the Aspartyl-tRNA synthetase. This enzyme interacting with differentiation-related gene 1 protein has a growth inhibitory role on cellular proliferation [51].

Taking into account the functional roles attributed to these proteins, it cannot be excluded that they are related to the molecular mechanism of WJCs *in vitro *self-renewal and multipotency.

## Conclusion

In this report we describe an extensive study of human WJCs proteome profiling. Several important proteins, including shootin 1, adenylate kinases 5 isoenzyme and plasminogen activator inhibitor 2, are no longer expressed after the early stage of cellular *in vitro *replication. This is probably correlated with the gradual reduction of their staminal multipotency. In addition, at the end of the cellular expansion, new proteins probably involved in the impairment of cellular survival during replication and differentiation time were identified. These proteins could be related to the biological cellular mechanism occurring in the cellular WJCs *in vitro *senescence.

Further investigation will be needed to elucidate biological mechanisms involved in maintaining active proliferation and maximal cellular plasticity in order to optimize *in vitro *culturing procedure.

WJCs obtained from the Wharton jelly umbilical cord therefore appear to be an inexpensive biological source for the isolation of stem cells thus circumventing the ethical constraint that arises from the use of the embryonic tissue.

In this context, this study carried out to increase the knowledge about the molecular features of WJCs, may give a great contribution to the field of regenerative medicine.

## Abbreviations

WJCs: Wharton's jelly cells; CFU-Fs: colony-forming unit-fibroblasts; MSCs: mesenchymal stem cells; hESCs: human embryonic stem cells; ANX1: annexin 1; ANX2: annexin 2; HSP27 b: heat shock protein 27 b; TUFM: elongation factor Tu; IDH1: isocitrate dehydrogenase; PRDX1: peroxiredoxin 1; PRDX2: peroxiredoxin 2; PRDX6: peroxiredoxin 6; SHOT1: shootin 1; KAD5: adenylate kinase 5; EIF3I: eukaryotic translation initiation factor 3 subunit I; PAI2: plasminogen activator inhibitor type-2; P4HA1: prolyl-4-hydroxylase subunit 1; P4HA2: prolyl-4-hydroxylase subunit 2; HIF: hypoxia-inducible factor; ERO1A: ERO1-like protein alpha; VIME: vimentin; ACTB: β-actin.

## Competing interests

The authors declare that they have no competing interests.

## Authors' contributions

SA: Conception and design, data analysis and interpretation, manuscript writing; MM: Provision of study material, cellular characterization, manuscript writing; FDG: 2DE sample preparation, proteomics experiments, image analysis, collection and/or assembly of data; LP: cellular isolation and culturing; SM: statistical evaluation, data analysis and interpretation; EE: statistical analysis; PL: Flow Cytometry immunophenotype characterization; GS: Provision of study material; SM: Final approval of manuscript; CDI: Financial support, data analysis and interpretation, manuscript writing, final approval of manuscript; All authors have read and approved the final manuscript.

## Supplementary Material

Additional file 1 **β-galactosidase staining at different passages *in vitro *expansion.** β-galactosidase staining at the 2^nd ^(A), 4^th ^(B), 8^th ^(C), 12^th ^(D) passage *in vitro *expansion. Examples of positive cells are indicated by arrows (magnification 100×). Click here for file

Additional file 2 **Scatter graphs of the % volume of about 2150 proteins between any of four WJCs lines at 2^nd^, 4^th^, 8^th ^and 12^th ^culture passages. **The correlation coefficient CC among any four lines has been reported. Click here for file

Additional file 3 Housekeeping proteins from WJCs. Click here for file
