# Supplementary figures and images for: Proteome analysis of human Wharton's jelly cells during in vitro expansion
Source: Proteome Sci. 2010 Mar 26;8:18. doi: 10.1186/1477-5956-8-18 (PMC2867805; doi:10.1186/1477-5956-8-18)

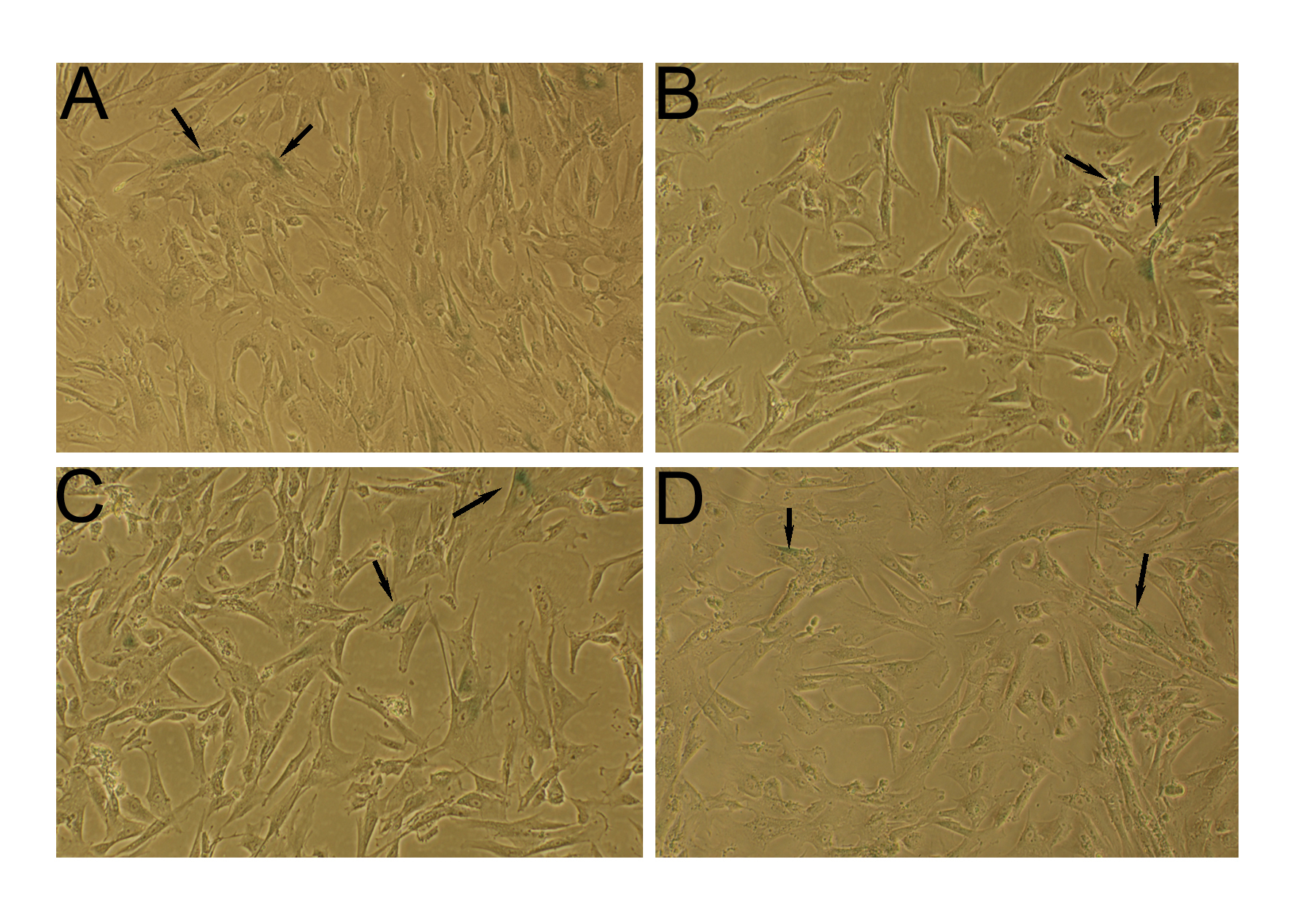

Supplement: Additional file 1 — β-galactosidase staining at different passages in vitro expansion. β-galactosidase staining at the 2nd (A), 4th (B), 8th (C), 12th (D) passage in vitro expansion. Examples of positive cells are indicated by arrows (magnification 100×). [file 1477-5956-8-18-S1.TIFF]

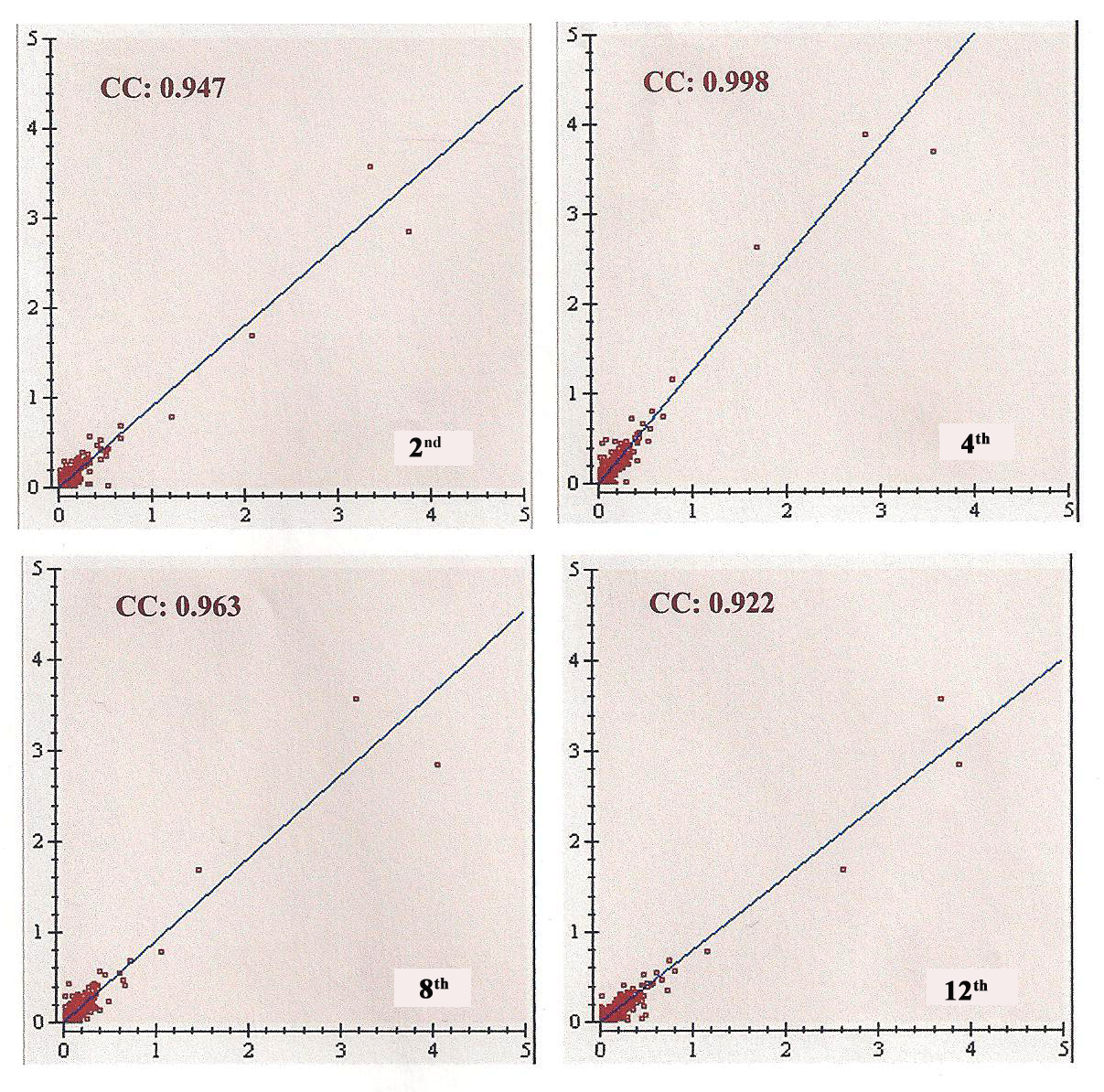

Supplement: Additional file 2 — Scatter graphs of the % volume of about 2150 proteins between any of four WJCs lines at 2nd, 4th, 8th and 12th culture passages. The correlation coefficient CC among any four lines has been reported. [file 1477-5956-8-18-S2.TIFF]
