# Supplementary material for: Proteome analysis of human Wharton's jelly cells during in vitro expansion
Source: Proteome Sci. 2010 Mar 26;8:18. doi: 10.1186/1477-5956-8-18 (PMC2867805; doi:10.1186/1477-5956-8-18)
Supplement: Additional file 3 — Housekeeping proteins from WJCs. [file 1477-5956-8-18-S3.DOC]

| **Additional file 3.** Housekeeping proteins from WJCs. | | |  |  |  |  |  |  |
| --- | --- | --- | --- | --- | --- | --- | --- | --- |
|  |  |  |  |  |  |  |  |  |
|  |  |  |  |  |  |  |  |  |
| Spot | Abbr. | Protein Name | Score^a^ | SC^b^ | Exp, | Exp, | Theo, | Theo, |
| ID | Name |  |  | % | *pI* | Mr(Da) | *pI* | Mr(Da) |
|  |  |  |  |  |  |  |  |  |
|  |  |  |  |  |  |  |  |  |
| 1112 | **ENPL** | Endoplasmin precursor | 335 | 49 | 4,80 | 91341 | 4,76 | 92696 |
| 1258 | **HS90A** | Heat shock protein HSP 90 B | 172 | 37 | 5,13 | 80223 | 4,94 | 85006 |
| 1059 | **GANAB** | Neutral alpha-glucosidase AB | 336 | 50 | 5,32 | 105091 | 5,74 | 107263 |
| 1101 | **PLOD2** | Procollagen dioxygenase 2 | 81 | 34 | 6,09 | 84300 | 6,24 | 85373 |
| 1851 | **PLST** | Plastin 3 | 176 | 44 | 5,41 | 71956 | 5,52 | 70904 |
| 2287 | **VIME** | Vimentin | 320 | 72 | 5,12 | 50213 | 5,06 | 53676 |
| 2868 | **ACTG** | Actin G | 235 | 65 | 5,07 | 44102 | 5,31 | 42108 |
| 2044 | **TCPZ** | T-complex protein 1 subunit zeta | 253 | 59 | 6,20 | 57113 | 6,23 | 58444 |
| 2586 | **ENOA** | Alpha-enolase | 183 | 54 | 6,98 | 45571 | 7,01 | 47481 |
| 2625 | **ARP3** | Actin-related protein 3 | 186 | 52 | 5,47 | 38935 | 5,61 | 47797 |
| 3316 | **RLAO** | 60S acidic ribosomal protein | 176 | 60 | 5,24 | 35484 | 5,51 | 34224 |
| 3414 | **PSDE** | Ptoteasome non ATPase | 66 | 30 | 5,95 | 36132 | 5,69 | 32417 |
| 3846 | **PGAM1** | Phosphoglycerate mutase 1 | 93 | 51 | 6,61 | 30933 | 6,68 | 26970 |
| 2965 | **RSSA** | 40S ribosomal protein SA | 175 | 46 | 4,83 | 39570 | 4,83 | 40369 |
| 3767 | **GSH0** | Glutamate-cysteine ligase regulatory subunit | 61 | 34 | 5,66 | 21254 | 5,74 | 27756 |
| 3692 | **PSME2** | Proteasome activator E2 | 132 | 56 | 5,27 | 28407 | 5,38 | 28587 |
| 3467 | **OTUB1** | Ubiquitin thioesterase | 110 | 54 | 4,82 | 37357 | 4,85 | 31493 |
| 3874 | **PSA5** | Proteasome subunit alpha type-5 | 140 | 61 | 4,67 | 25011 | 4,74 | 26565 |
| 4051 | **VBE2K** | Ubiquitin-conjugating enzyme E2 K | 79 | 39 | 5,32 | 23345 | 5,33 | 22507 |
| 4105 | **EIF3K** | Eukaryotic translation initiation factor 3 subunit K | 80 | 61 | 4,88 | 25790 | 4,81 | 25329 |
| 4329 | **NDKA** | Nucleoside diphosphate kinase A | 184 | 91 | 5,67 | 18935 | 5,74 | 19772 |
| 4439 | **IF5A1** | Eukaryotic translation initiation factor 3 subunit K | 76 | 58 | 5,07 | 12807 | 5,08 | 17049 |
| 4631 | **LEG1** | Galectin 1 | 162 | 74 | 5,22 | 16434 | 5,34 | 15048 |
| 4737 | **S100AB** | Protein S100-A11 | 90 | 52 | 6,47 | 18935 | 6,56 | 11847 |
| 2853 | **PPME1** | Protein phosphatase methylesterase | 100 | 41 | 5,34 | 42807 | 5,67 | 42687 |
| 1851 | **VINC** | Vinculin | 332 | 43 | 5,58 | 116790 | 5,50 | 124292 |
| 1607 | **SYG** | Glycil-tRNA synthase | 101 | 56 | 6,24 | 79599 | 5,65 | 76960 |
| 1772 | **ANXA6** | Annexin A6 | 268 | 62 | 5,36 | 76787 | 5,45 | 72640 |
| 1224 | **HSP7C** | Heat shock cognate 71 kDa | 227 | 59 | 5,37 | 71082 | 5,4 | 71222 |
| 1250 | **P3H3** | Prolyl 3-hydroxylase 3 | 106 | 33 | 5,48 | 71082 | 5,65 | 87223 |
| 1499 | **CALD1** | Caldesmon | 156 | 29 | 5,86 | 74243 | 6,52 | 79697 |
|  |  |  |  |  |  |  |  |  |
|  |  |  |  |  |  |  |  |  |

| **Supplemental table 1.** Continued | | |  |  |  |  |  |  |
| --- | --- | --- | --- | --- | --- | --- | --- | --- |
|  |  |  |  |  |  |  |  |  |
|  |  |  |  |  |  |  |  |  |
| Spot | Abbr. | Protein Name | Score^a^ | SC^b^ | Exp. | Exp. | Theo. | Theo, |
| ID | Name |  |  | % | *pI* | Mr(Da) | *pI* | Mr(Da) |
|  |  |  |  |  |  |  |  |  |
|  |  |  |  |  |  |  |  |  |
| 1559 | **GRP78** | Glucose related protein 78 | 163 | 36 | 5,27 | 72044 | 5,22 | 77375 |
| 2153 | **PDIA1** | Ptotein disulfide isomerase A1 | 280 | 61 | 4,72 | 51092 | 4,83 | 58044 |
| 2428 | **TBB5** | Tubulin | 88 | 25 | 5,09 | 55446 | 4,91 | 51777 |
| 1934 | **TCPA** | T-complex protein 1 | 188 | 46 | 5,63 | 63141 | 5,99 | 63766 |
| 1862 | **LKHA4** | Leukotriene A-4 | 143 | 51 | 6,32 | 75883 | 6,40 | 70904 |
| 1237 | **NIBL1** | Nibal- Like Protein 1 | 97 | 35 | 5,62 | 86152 | 5,56 | 87632 |
| 1353 | **KU86** | ATP-Dependent DNA helicase 2 | 136 | 40 | 5,53 | 83619 | 5,52 | 83534 |
| 2041 | **TCPA** | T-complex protein 1 subunit alpha | 257 | 61 | 6,07 | 57771 | 5,98 | 58843 |
| 2204 | **TXND1** | Thioredoxin | 114 | 39 | 5,79 | 58458 | 6,01 | 58181 |
| 2274 | **PDIA3** | Protein Disulfide Isomerase 3 | 246 | 51 | 5,64 | 53285 | 5,67 | 54059 |
| 2346 | **SYWC** | Tryptophanyl-tRNA synthetase | 105 | 46 | 4,59 | 50414 | 6,25 | 53405 |
| 2493 | **RHG01** | Rho GTPase-activating protein 1 | 116 | 48 | 5,78 | 52259 | 5,85 | 50458 |
| 2582 | **SNX6** | Sortin-nexin 6 | 109 | 40 | 6,25 | 44386 | 6,17 | 48794 |
| 2463 | **MPPA** | Mitochondrial-processing peptidase subunit alpha | 94 | 43 | 5,72 | 51202 | 5,79 | 51577 |
| 2354 | **SYWC** | Tryptophanyl-tRNA synthetase, cytoplasmic | **116** | **46** | 5,43 | 53674 | 5,83 | 53474 |
| 1915 | **AGM1** | Phosphoacetylglucosamine mutase | 102 | 34 | 6,05 | 65260 | 5,81 | 66986 |
| 2604 | **ENOA** | Alpha-enolase | 102 | 44 | 6,04 | 47179 | 6,04 | 48293 |
| 2705 | **VAT1** | Synaptic vesicle membrane protein VAT-1 | 89 | 41 | 5,60 | 43980 | 5,77 | 45489 |
| 2700 | **PRS7** | 26S protease regulatory subunit 7 | 227 | 65 | 5,69 | 45383 | 5,74 | 45995 |
| 2682 | **GDIB** | Rab GDP dissociation inhibitor beta | 60 | 45 | 5,63 | 47134 | 5,65 | 46507 |
| 2590 | **ENOA** | Alpha-enolase | 127 | 48 | 6,35 | 48522 | 6,23 | 48231 |
| 2946 | **SEPT2** | Septin 2 | 168 | 63 | 6,36 | 39846 | 6,18 | 40760 |
| 3023 | **UBCP1** | Ubiquitin-like domain-containing CTD phosph. 1 | 128 | 42 | 5,77 | 39376 | 5,91 | 39502 |
| 3133 | **GIPC1** | PDZ domain-containing protein GIPC1 | 128 | 42 | 4,79 | 35814 | 5,77 | 37552 |
| 3085 | **DCUP** | Uroporphyrinogen decarboxylase | 141 | 49 | 5,10 | 38711 | 5,75 | 38422 |
| 3179 | **BIEA** | Biliverdin reductase | 206 | 48 | 5,82 | 34763 | 6,00 | 36746 |
| 3081 | **EIF3H** | Eukaryotic translation initiation factor 3 subunit H | 81 | 49 | 5,42 | 36717 | 6,24 | 38515 |
| 2964 | **FAAA** | Fumaryl Acetoacetase | 99 | 38 | 6,47 | 39421 | 6,86 | 40515 |
| 3257 | **ANXA1** | Annexin A1 | 272 | 67 | 6,62 | 33979 | 6,47 | 34681 |
| 3043 | **TWF2** | Twinfilin 2 | 99 | 39 | 5,68 | 35555 | 6,86 | 38795 |
| 3169 | **AK1A1** | Aldo-keto reductase family 1 member A1 | 161 | 48 | 6,76 | 33287 | 6,74 | 36969 |
| 2960 | **ACTB** | Actin, cytoplasmic 1 (Beta-actin) | 207 | 55 | 5,56 | 40073 | 5,66 | 40662 |
| 3016 | **PSD13** | 26S proteasome non-ATPase regulatory subunit 13 | 179 | 53 | 5,58 | 39875 | 5,63 | 39502 |
|  |  |  |  |  |  |  |  |  |
|  |  |  |  |  |  |  |  |  |

| **Supplemental table 1.** Continued | | |  |  |  |  |  |  |
| --- | --- | --- | --- | --- | --- | --- | --- | --- |
|  |  |  |  |  |  |  |  |  |
|  |  |  |  |  |  |  |  |  |
| Spot | Abbr. | Protein Name | Score^a^ | SC^b^ | Exp, | Exp, | Theo, | Theo, |
| ID | Name |  |  | % | *pI* | Mr(Da) | *pI* | Mr(Da) |
|  |  |  |  |  |  |  |  |  |

| 3124 | **SIAS** | Sialic acid synthase | 68 | 27 | 6,99 | 37643 | 7,01 | 37598 |
| --- | --- | --- | --- | --- | --- | --- | --- | --- |
| 3660 | **PSA1** | Proteasome subunit alpha type-1 | 99 | 47 | 4,83 | 38054 | 6,23 | 28913 |
| 3737 | **GSTO1** | Glutathione S-transferase Omega 1 | 91 | 40 | 4,82 | 29218 | 5,52 | 28137 |
| 3832 | **ERP29** | Endoplasmic reticulum protein ERp29 | 102 | 45 | 5,39 | 27765 | 5,73 | 27031 |
| 3905 | **PRDX6** | Peroxiredoxin 6 | 187 | 66 | 6,26 | 25147 | 6,24 | 25757 |
| 3925 | **TPIS** | Triosephosphate isomerase | 82 | 51 | 5,80 | 28231 | 5,98 | 25507 |
| 3818 | **PGAM1** | Phosphoglycerate mutase 1 | 107 | 43 | 6,25 | 30719 | 6,23 | 27154 |
| 4020 | **PSB3** | Proteasome subunit beta type-3 | 102 | 50 | 5,86 | 22415 | 5,81 | 22950 |
| 4075 | **PARK7** | Protein DJ | 128 | 81 | 5,11 | 40126 | 5,78 | 23809 |
| 4042 | **BAG2** | BAG family molecular chaperone regulator 2 | 102 | 63 | 6,18 | 24723 | 6,24 | 22802 |
| 4036 | **PRDX3** | Peroxiredoxin 3 | 72 | 37 | 5,49 | 22208 | 5,52 | 22570 |
| 4025 | **PSD10** | 26 S proteasome subunit 2 | 109 | 50 | 5,34 | 27962 | 5,66 | 22988 |
| 4094 | **ABHEB** | Abhydrolase domain-containing protein 14B | 88 | 51 | 5,54 | 20515 | 5,75 | 24696 |
| 3881 | **IF4E** | Eukaryotic translation initiation factor 4E | 73 | 36 | 6,24 | 32029 | 5,73 | 26522 |
| 3992 | **GRB2** | Growth factor receptor-bound protein 2 | 136 | 46 | 5,80 | 33369 | 5,95 | 24019 |
| 3572 | **ANXA4** | Annexin A4 | 241 | 59 | 5,48 | 43221 | 5,66 | 30028 |
| 4365 | **HBB** | Beta globin | 67 | 57 | 5,94 | 18860 | 5,67 | 18590 |
| 4331 | **HSPB6** | Heat shock protein beta-6 | 66 | 51 | 5,93 | 19348 | 5,55 | 19748 |
| 4360 | **ARP5L** | Actin-related protein subunit 5-like protein | 71 | 50 | 5,81 | 18519 | 5,53 | 18901 |
| 4416 | **ACTG1** | Actin, cytoplasmic 2 | 104 | 47 | 5,33 | 17228 | 5,32 | 17375 |
| 4421 | **ARPC5** | Actin-related protein subunit 5 | 121 | 69 | 5,40 | 19837 | 5,50 | 17314 |
| 3939 | **UCHL1** | Ubiquitin carboxyl-terminal hydrolase isozyme | 141 | 68 | 5,46 | 24382 | 5,40 | 24651 |
| 4612 | **PHP14** | 14 kDa phosphohistidine phosphatase | 70 | 52 | 5,74 | 15691 | 5,44 | 15269 |
| 4696 | **NTF2** | Nuclear trasport factor 2 | 73 | 69 | 4,89 | 12883 | 4,83 | 13188 |
| 4517 | **MYL6** | Myosin light polypeptide 6 | 108 | 58 | 4,95 | 16833 | 4,60 | 16082 |
| 4319 | **MLY9** | Myosin regulatory light polypeptide 9 | 114 | 62 | 4,77 | 19422 | 4,85 | 19818 |
| 4095 | **LGUL** | Lactoylglutathione lyase | 67 | 36 | 5,10 | 24112 | 4,99 | 24835 |
| 3892 | **CPNS1** | Calpain small subunit 1 | 104 | 43 | 5,08 | 82088 | 4,92 | 26179 |
| 3549 | **ANXA5** | Annexin A5 | 180 | 71 | 5,29 | 89918 | 4,92 | 30210 |
| 3729 | **ACTG** | Actin G | 69 | 32 | 4,78 | 26445 | 4,90 | 28052 |
| 3659 | **EF1B** | Elongation factor 1-beta | 62 | 29 | 4,66 | 28316 | 4,63 | 28891 |

|  |  |  |  |  |  |  |  |  |
| --- | --- | --- | --- | --- | --- | --- | --- | --- |

| **Supplemental table 1.** Continued | | |  |  |  |  |  |  |
| --- | --- | --- | --- | --- | --- | --- | --- | --- |
|  |  |  |  |  |  |  |  |  |
|  |  |  |  |  |  |  |  |  |
| Spot | Abbr. | Protein Name | Score^a^ | SC^b^ | Exp, | Exp, | Theo, | Theo, |
| ID | Name |  |  | % | *pI* | Mr(Da) | *pI* | Mr(Da) |
|  |  |  |  |  |  |  |  |  |

| 3803 | **ACTB** | Actin B | 79 | 36 | 5,18 | 26434 | 5,32 | 27216 |
| --- | --- | --- | --- | --- | --- | --- | --- | --- |
| 3669 | **CLIC1** | Chloride intracellular channel protein 1 | 85 | 55 | 5,74 | 25897 | 5,17 | 28804 |
| 3805 | **KCP1** | 14-3-3 zeta/delta protein (variant) | 237 | 75 | 4,64 | 27833 | 4,76 | 21236 |
| 3595 | **TPM4** | Tropomyosin 4 | 119 | 49 | 4,72 | 61157 | 4,72 | 29510 |
| 3501 | **TPM1** | Tropomyosin 1 | 62 | 33 | 4,84 | 30164 | 4,75 | 30880 |
| 3375 | **EF1D** | Elongation factor 1-delta | 64 | 53 | 4,82 | 31531 | 4,86 | 33009 |
| 3227 | **TPM2** | Tropomyosin 2 | 124 | 35 | 4,80 | 35055 | 4,70 | 35229 |
| 3233 | **NPM** | Nucleophosmin 1 | 55 | 28 | 4,83 | 34502 | 4,79 | 35441 |
| 2988 | **K1C19** | Keratin C19 | 336 | 67 | 5,16 | 38422 | 5,28 | 40174 |
| 2766 | **PPIR7** | Protein phosphatase 1 regulatory subunit 7 | 87 | 43 | 5,03 | 44585 | 4,81 | 44433 |
| 3565 | **VIME** | Vimentin | 99 | 32 | 5,04 | 29013 | 5,34 | 30141 |
| 3744 | **PHB** | Prohibitin | 126 | 65 | 5,44 | 30742 | 5,45 | 27988 |
| 3859 | **NDUS2** | NADH dehydrogenase iron-sulfur protein 2 | 135 | 48 | 5,70 | 27296 | 5,50 | 26585 |
| 3733 | **GSTO1** | Glutathione S-transferase Omega 1 | 100 | 44 | 5,49 | 31510 | 5,69 | 28158 |
| 4040 | **GSTP1** | Glutathione S-transferase Pi 1 | 185 | 60 | 5,48 | 23995 | 5,49 | 22954 |
| 3977 | **ORN** | Oligoribonuclease | 135 | 58 | 5,45 | 22746 | 5,49 | 24175 |
| 3800 | **VIME** | Vimentin | 204 | 42 | 5,63 | 23884 | 5,50 | 27505 |
| 3745 | **PA1B2** | Platelet activating factor 1 subunit B2 | 116 | 50 | 5,66 | 31254 | 5,58 | 27988 |
| 4624 | **LEG1** | Galectin 1 | 62 | 64 | 4,67 | 15107 | 4,46 | 15136 |
| 4543 | **COTL1** | Coactosin-like protein | 80 | 40 | 5,35 | 16132 | 5,32 | 15883 |
| 4515 | **UBE2N** | Ubiquitin-conjugating enzyme E2 N | 115 | 59 | 6,01 | 18933 | 5,94 | 16200 |
| 4379 | **MRLC3** | Myosin regulatory light chain 3 | 96 | 63 | 4,44 | 15484 | 4,77 | 18436 |
| 3559 | **MVP** | Major Valut Protein | 151 | 40 | 5,65 | 17732 | 5,50 | 17855 |
| 4680 | **S10AD** | Protein S100-A13 | 35 | 29 | 5,58 | 13668 | 5,91 | 11464 |
| 4653 | **LEG1** | Galectin 1 | 73 | 73 | 5,31 | 14560 | 5,34 | 15048 |
| 4565 | **ACTG** | Actin, cytoplasmic 2 | 103 | 36 | 5,82 | 15699 | 5,24 | 18724 |
| 4556 | **ACTB** | Actin, cytoplasmic 1 | 154 | 68 | 6,23 | 15745 | 5,04 | 14955 |
| 4528 | **CS010** | UPF0556 protein C19orf10 | 56 | 23 | 6,64 | 16105 | 6,20 | 18897 |
| 4259 | **MGN** | Protein mago nashi homolog | 78 | 46 | 5,52 | 21584 | 5,74 | 17210 |
| 4350 | **PK** | Piruvate Kinase | 64 | 49 | 6,87 | 56036 | 7,95 | 58470 |
| 4406 | **STMN1** | Stathmin | 64 | 49 | 5,64 | 17708 | 5,76 | 17292 |

|  |  |  |  |  |  |  |  |  |
| --- | --- | --- | --- | --- | --- | --- | --- | --- |

| **Supplemental table 1.** Continued | | |  |  |  |  |  |  |
| --- | --- | --- | --- | --- | --- | --- | --- | --- |
|  |  |  |  |  |  |  |  |  |
|  |  |  |  |  |  |  |  |  |
| Spot | Abbr. | Protein Name | Score^a^ | SC^b^ | Exp, | Exp, | Theo, | Theo, |
| ID | Name |  |  | % | *pI* | Mr(Da) | *pI* | Mr(Da) |
|  |  |  |  |  |  |  |  |  |

| 4208 | **ACTG** | Actin, cytoplasmic 2 | 121 | 43 | 5,39 | 22820 | 5,20 | 28478 |
| --- | --- | --- | --- | --- | --- | --- | --- | --- |
| 4109 | **PRDX2** | Peroxiredoxin-2 | 102 | 53 | 5,46 | 24853 | 5,66 | 22046 |
| 4126 | **PSB2** | Proteasome subunit beta type-2 | 103 | 47 | 7,16 | 25031 | 6,51 | 22993 |
| 4044 | **NDUV2** | NADH dehydrogenase flavoprotein 2 | 86 | 46 | 5,82 | 22728 | 8,22 | 27659 |
| 4035 | **ACTG** | Actin, cytoplasmic 2 | 68 | 37 | 5,55 | 22839 | 5,65 | 26147 |
| 3910 | **PRDX6** | Peroxiredoxin-6 | 214 | 76 | 6,37 | 25799 | 6,00 | 25133 |
| 3877 | **PRDX4** | Peroxiredoxin-4 | 290 | 68 | 5,61 | 26010 | 5,86 | 30749 |
| 3830 | **ESR1** | Nuclear receptor subfamily 1 | 62 | 24 | 5,63 | 27134 | 5,44 | 31315 |
| 4060 | **TCTP** | Translationally-controlled tumor protein | 60 | 53 | 4,78 | 23676 | 5,11 | 22787 |
| 3889 | **IF6** | Eukaryotic translation initiation factor 6 | 71 | 59 | 4,64 | 26350 | 4,56 | 27095 |
| 3896 | **UCHL3** | Ubiquitin carboxyl-terminal hydrolase isozyme L3 | 103 | 66 | 4,83 | 26179 | 4,84 | 26337 |
| 3819 | **ACTC** | Alpha-cardiac actin | 99 | 31 | 5,27 | 27052 | 4,88 | 30498 |
| 3631 | **PSME3** | Proteasome activator complex subunit 3 | 80 | 50 | 5,65 | 29398 | 5,69 | 29602 |
| 3514 | **PIPNB** | Phosphatidylinositol transfer protein beta isoform | 75 | 32 | 6,24 | 30833 | 6,41 | 31805 |
| 3418 | **AIDA** | Axin interactor, dorsalization associated protein | 115 | 49 | 6,09 | 32496 | 6,13 | 35172 |
| 3226 | **PSD7** | 26S proteasome non-ATPase regulatory subunit 7 | 75 | 43 | 7,16 | 35699 | 6,29 | 37060 |
| 2936 | **RCN3** | Reticulocalbin-3 | 119 | 37 | 4,73 | 40858 | 4,74 | 37470 |
| 3029 | **RCN1** | Reticulocalbin-1 | 91 | 35 | 4,74 | 39455 | 4,86 | 38886 |
| 2654 | **RINI** | Ribonuclease inhibitor | 182 | 62 | 4,74 | 46765 | 4,74 | 51209 |
| 2531 | **ATPB** | ATP synthase subunit beta, mitochondrial | 132 | 42 | 5,29 | 49747 | 5,26 | 56525 |
| 2580 | **PDIA6** | Protein disulfide-isomerase A6 | 155 | 46 | 5,08 | 48857 | 4,95 | 48490 |
| 2736 | **M6PBP** | Mannose-6-phosphate receptor-binding protein 1 | 170 | 50 | 5,25 | 44803 | 5,30 | 47189 |
| 2488 | **HNRH1** | Heterogeneous nuclear ribonucleoprotein H | 129 | 59 | 5,74 | 50393 | 5,89 | 49484 |
| 3122 | **ARFP2** | Arfaptin-2 | 51 | 31 | 5,68 | 37552 | 5,72 | 37946 |
| 3028 | **CAPG** | Macrophage-capping protein | 84 | 48 | 6,52 | 39407 | 5,88 | 38779 |
| 3292 | **ADPPT** | Dehydrogenase-phosphopantetheinyl transferase | 76 | 43 | 5,67 | 34806 | 6,35 | 35981 |
| 3610 | **TSNAX** | Translin-associated protein X | 150 | 64 | 5,77 | 29622 | 6,10 | 33206 |
| 2816 | **OAT** | Ornithine aminotransferase, mitochondrial | 127 | 47 | 5,86 | 43581 | 6,57 | 48846 |
| 2763 | **CBPA4** | Carboxypeptidase A4 | 157 | 50 | 6,61 | 43884 | 6,23 | 47550 |
| 2691 | **PSD11** | 26S proteasome non-ATPase regulatory subunit 11 | 230 | 61 | 6,06 | 46186 | 6,08 | 47719 |
| 3071 | **ROAA** | Heterogeneous nuclear ribonucleoprotein A/B | 48 | 22 | 6,62 | 38608 | 8,22 | 36316 |

|  |  |  |  |  |  |  |  |  |
| --- | --- | --- | --- | --- | --- | --- | --- | --- |

| **Supplemental table 1.** Continued | | |  |  |  |  |  |  |
| --- | --- | --- | --- | --- | --- | --- | --- | --- |
|  |  |  |  |  |  |  |  |  |
|  |  |  |  |  |  |  |  |  |
| Spot | Abbr. | Protein Name | Score^a^ | SC^b^ | Exp, | Exp, | Theo, | Theo, |
| ID | Name |  |  | % | *pI* | Mr(Da) | *pI* | Mr(Da) |
|  |  |  |  |  |  |  |  |  |

| 3327 | **CK068** | UPF0696 protein C11orf68 | 90 | 44 | 5,82 | 34183 | 5,41 | 27537 |
| --- | --- | --- | --- | --- | --- | --- | --- | --- |
| 3185 | **PRDBP** | Protein kinase C delta-binding protein | 82 | 41 | 5,66 | 36570 | 5,86 | 27609 |
| 2810 | **EFTU** | Elongation factor Tu | 103 | 44 | 6,90 | 43102 | 7,26 | 49852 |
| 2679 | **PA2G4** | Proliferation-associated protein 2G4 | 140 | 48 | 6,24 | 46442 | 6,13 | 44101 |
| 2671 | **TS101** | Tumor susceptibility gene 101 protein | 57 | 43 | 5,58 | 46765 | 6,06 | 44048 |
| 2418 | **AL1B1** | Aldehyde dehydrogenase X, mitochondrial | 118 | 35 | 6,24 | 52247 | 6,36 | 57658 |
| 2465 | **RUVB1** | RuvB-like 1 | 121 | 52 | 6,23 | 51444 | 6,02 | 50538 |
| 2887 | **PNM6B** | Paraneoplastic antigen-like protein 6B | 48 | 22 | 6,38 | 42007 | 5,31 | 44378 |
| 2485 | **SEP11** | Septin-11 | 138 | 54 | 6,84 | 50523 | 6,36 | 49652 |
| 2464 | **RUVB1** | RuvB-like 1 | 117 | 50 | 6,16 | 51577 | 6,02 | 50538 |
| 2297 | **PRP19** | Pre-mRNA-processing factor 19 | 56 | 17 | 6,24 | 54316 | 6,14 | 55603 |
| 2064 | **DPYL3** | Dihydropyrimidinase-related protein 3 | 273 | 63 | 6,24 | 59650 | 6,04 | 62323 |
| 2077 | **UAP1** | UDP-N-acetylhexosamine pyrophosphorylase | 166 | 43 | 5,53 | 60330 | 5,92 | 59131 |
| 1942 | **AGM1** | Phosphoacetylglucosamine mutase | 70 | 28 | 6,18 | 64008 | 5,84 | 60270 |
| 1964 | **MPP5** | MAGUK p55 subfamily member 5 | 102 | 22 | 5,65 | 63045 | 5,77 | 77531 |
| 1735 | **FKBP9** | FK506-binding protein 9 | 100 | 30 | 4,83 | 73920 | 5,91 | 63500 |
| 1703 | **VIME** | Vimentin | 116 | 41 | 5,06 | 74819 | 5,06 | 53676 |
| 747 | **ENPL** | Endoplasmin | 144 | 33 | 4,76 | 96701 | 4,76 | 92693 |
| 552 | **VINC** | Vinculin | 67 | 17 | 6,43 | 110202 | 5,50 | 124292 |
| 1416 | **PLOD3** | Procollagen-lysine,2-oxoglutarate 5-dioxygenase 3 | 288 | 51 | 5,82 | 82308 | 5,69 | 85302 |
| 1476 | **EZRI** | Ezrin | 186 | 48 | 5,96 | 80558 | 5,94 | 69484 |
| 1217 | **P3H3** | Prolyl 3-hydroxylase 3 | 189 | 37 | 5,65 | 88250 | 5,93 | 82584 |
| 1737 | **SCFD1** | Sec1 family domain-containing protein 1 | 156 | 28 | 5,74 | 74418 | 5,89 | 72676 |
| 1362 | **IMMT** | Mitochondrial inner membrane protein | 55 | 19 | 6,21 | 83759 | 6,08 | 84025 |
| 2001 | **MAOX** | NADP-dependent malic enzyme | 85 | 34 | 5,72 | 61628 | 5,79 | 64679 |
| 3813 | **6PGL** | 6-phosphogluconolactonase | 98 | 56 | 5,71 | 27236 | 5,70 | 27815 |

|  |  |  |  |  |  |  |  |  |
| --- | --- | --- | --- | --- | --- | --- | --- | --- |

Spot ID is the identification number assigned to each spot detected by image analysis; abbreviated and protein names correspond to the nomenclature used in the Swiss-Prot database.

1. Score is -10*Log(P), where P is the probability that the observed match is a random event, it is based on Swiss-Prot database using the MASCOT searching program as MALDI-TOF data.
2. Sequence coverage means the ratio of portion sequence covered by matched peptide to the full length of the protein sequence.
